# External Validation of a Nomogram and Risk Grouping System for Predicting Individual Prognosis of Patients With Medulloblastoma

**DOI:** 10.3389/fphar.2020.590348

**Published:** 2020-11-11

**Authors:** Chengcheng Guo, Dunchen Yao, Xiaoping Lin, He Huang, Ji Zhang, Fuhua Lin, Yonggao Mou, Qunying Yang

**Affiliations:** ^1^ Department of Neurosurgery/Neuro-Oncology, Sun Yat-Sen University Cancer Center, Collaborative Innovation Center for Cancer Medicine, State Key Laboratory of Oncology in South China, Guangzhou, China; ^2^ Department of Radiation Oncology, Sun Yat-Sen University Cancer Center, Collaborative Innovation Center for Cancer Medicine, State Key Laboratory of Oncology in South China, Guangzhou, China; ^3^D epartment of Nuclear Medicine, Sun Yat-Sen University Cancer Center, Collaborative Innovation Center for Cancer Medicine, State Key Laboratory of Oncology in South China, Guangzhou, China; ^4^ Department of Medical Oncology, Sun Yat-Sen University Cancer Center, Collaborative Innovation Center for Cancer Medicine, State Key Laboratory of Oncology in South China, Guangzhou, China

**Keywords:** neuroepithelial tumors, training cohort, validation cohort, overall survival, Surveillance, Epidemiology, and End Results

## Abstract

**Background:** Medulloblastoma (MB) is one of the most malignant neuroepithelial tumors in the central nervous system. This study aimed to establish an effective prognostic nomogram and risk grouping system for predicting overall survival (OS) of patients with MB.

**Materials and Methods:** The nomogram was constructed based on data from the database of Surveillance, Epidemiology, and End Results (SEER). This database consisted of 2,824 patients with medulloblastoma and was used as the training cohort. The data of another additional 161 patients treated at the Sun Yat-sen University Cancer Center (SYSUCC) were used as the external validation cohort. Cox regression analysis was used to select independent prognostic factors. Concordance index (C-index) and calibration curve were used to predict the prognostic effect of the nomogram for overall survival.

**Results:** In the training cohort, Cox regression analyses showed that the prognostic factors included histopathology, surgery, radiotherapy, chemotherapy, tumor size, dissemination, and age at diagnosis. The internal and external validated C-indexes were 0.681 and 0.644, respectively. Calibration curves showed that the nomogram was able to predict 1-, 3-, and 5-year OS for patients with MB precisely. Using the training cohort, a risk grouping system was built, which could perfectly classify patients into four risk nomogroups with a 5-year survival rate of 83.9%, 76.5%, 64.5%, and 46.8%, respectively.

**Conclusion:** We built and validated a nomogram and risk grouping system that can provide individual prediction of OS and distinguish MB patients from different risk groups. This nomogram and risk grouping system could help clinicians making better treatment plan and prognostic assessment.

## Introduction

Medulloblastoma (MB) is one of the most malignant brain tumors in children (Khanna et al., 2017; Leece et al., 2017). Current standard treatment consists of 36 Gy of craniospinal irradiation supplemented with 18–20 Gy of local irradiation (total dose of 54–56 Gy) and adjuvant chemotherapy after surgery (Sirachainan et al., 2011). Although most of the MB cases are curable, around 35% of patients in the average-risk group and 50% in the poor-risk group would relapse in 5 years (Sirachainan et al., 2011; Tamayo et al., 2011). As a result, it is essential to set up an effective prognostic model for accurately identifying such patients for better treatment and surveillance evaluation (He et al., 2018; Wu et al., 2019). In regard to pediatric MB, clinical staging systems including the extent of the tumor, the tumor histology, and age of patients have long been considered as the most powerful stratification system to classify patients into different prognostic risk subgroups (Korshunov et al., 2010; Sirachainan et al., 2011). Children aged older than 3 years had a superior prognosis. As for molecular classification in the 2016 WHO classification, four principal molecular subgroups based on transcriptome and methylome profiling, including Wingless (WNT), Sonic Hedgehog (SHH), group 3 (G3), and group 4 (G4), were identified to be significantly better correlated with different prognosis than traditional subtypes (Taylor et al., 2012; Louis et al., 2016; Liu et al., 2017; Vo et al., 2018). However, the studies on prognosis of MB are still needed to confirm the precise pattern of prognosis.

A nomogram was a graphical mathematical algorithm of a statistical predictive model (Kattan et al., 1998) to generate a precise prediction based on the evaluation of important factors for estimating the conditional risk of disease outcomes (Wang et al., 2018). There have been a number of studies using nomograms for prognosis of cancers such as bladder, colorectal, lung, nasopharyngeal, and breast cancer (Mariani et al., 2005; Iasonos et al., 2008; Gold et al., 2009; Valentini et al., 2011; Wang et al., 2013; Chen et al., 2015; OuYang et al., 2018; Tang et al., 2019). However, nomograms for predicting prognosis of MB are limited.

In this study, we aimed to establish a nomogram for predicting the prognosis of MB from two different patient populations and to externally validate it.

## Materials and Methods

### Patients and Study Variable

The data of MB patients from the database of the Surveillance, Epidemiology, and End Results (SEER) were extracted using the following specifications: “Incidence—SEER 18 Registries Custom Data (with additional treatment fields), November 2016 Submission (1973–2014 varying).” Inclusion criteria for patient selection included: 1) pathologically proven diagnosis of medulloblastoma; 2) according to the third edition of International Classification of Diseases for Oncology (ICD-O-3) to identify cases of MB, and the following ICD-O-3 codes for histopathology were chosen: 9,470/3 medulloblastoma, NOS; 9,471/3 desmoplastic nodular medulloblastoma; 9,474/3 large cell medulloblastoma; and 3) known survival status and survival time. These data were used as a training cohort.

In addition, to validate the nomogram, we searched the Sun Yat-sen University Cancer Center (SYSUCC) database for patients diagnosed with medulloblastoma between 1971 and 2018. The inclusion criteria for case selection were: 1) pathologically confirmed diagnosis of medulloblastoma; 2) no prior anticancer therapy; and 3) complete clinicopathologic characteristics, therapeutic records, and complete follow-up data.

### Definition of Variables

Considering some incomplete data variables from the SEER, we redefine the variables for developing the nomogram. In our study, surgery was categorized as: total resection (TR), subtotal resection (SR), surgery, NOS, and no evidence. Histopathology was categorized as: medulloblastoma, NOS, desmoplastic nodular medulloblastoma, and large cell medulloblastoma. For objectively assessing the prognostic value of age and tumor size for MB patients, the patients were stratified into two categories, namely ≤3 years, >3 years; or ≤6.5 cm, >6.5 cm, and/or unknown, using the X-tile (https://x-tile.software.informer.com, Yale School of Medicine, New Haven, CT, United States) software for obtaining the best cutoff value. Moreover, dissemination of tumor cells was defined as findings of distant metastasis based on radiographical images or tumor cells in cerebral spinal fluid (CSF).

### Construction and Validation of the Nomogram

We identified independent prognostic factors by univariate and multivariate Cox regression model in the training cohort, which were then used to construct the nomogram to predict the 1-, 3-, and 5-year overall survival (OS) rates. Based on the contribution of each factor for the outcome in the model, the nomogram can express the relationship between various variables in the model according to drawing a line segment with certain proportion on the same plane, which transformed the complex regression equation into graphic visualization, and made the results of the prediction model more reifiable and convenient for clinician and patients.

The performance of the nomogram including its discrimination and calibration was validated using the SYSUCC cohort as external validation. Discrimination was assessed using a concordance index (C-index), and the larger the C-index, the more accurate the nomogram. Calibration was performed by comparing predicted survival rate actual survival rate determined using Kaplan–Meier analysis for 1-, 3-, and 5-year overall survival. In addition, we did bootstrap 1,000 resamples to verify the accuracy of the nomogram in the training cohort and external validation cohort from SYSUCC database.

### Risk Grouping

In this study, we defined risk as the harmful factors for prognosis of MB patients. To develop the risk grouping to assess the prognosis of MB patients, we divided patients from the training cohort into four prognostic groups according to the total points of each patient. Furthermore, we used Kaplan–Meier curves to compare the prognosis of patients in the different nomogroups.

### Statistical Analysis

All statistical analyses were performed by the statistical software package of SPSS for Windows, version 22.0 (IBM Corporation, Armonk, NY, United States), *p*-values were two-sided, and *p*-values <0.05 were considered as statistically significant. The nomogram was constructed by R 3.5.1 (http://www.r-project.org) and all diagrams of this study were drawn by GraphPad Prism 8 (https://www.graphpad.com/scientific-software/prism/).

## Results

### Clinicopathological Characteristics of Training and Validation Cohort

**FIGURE 1:**
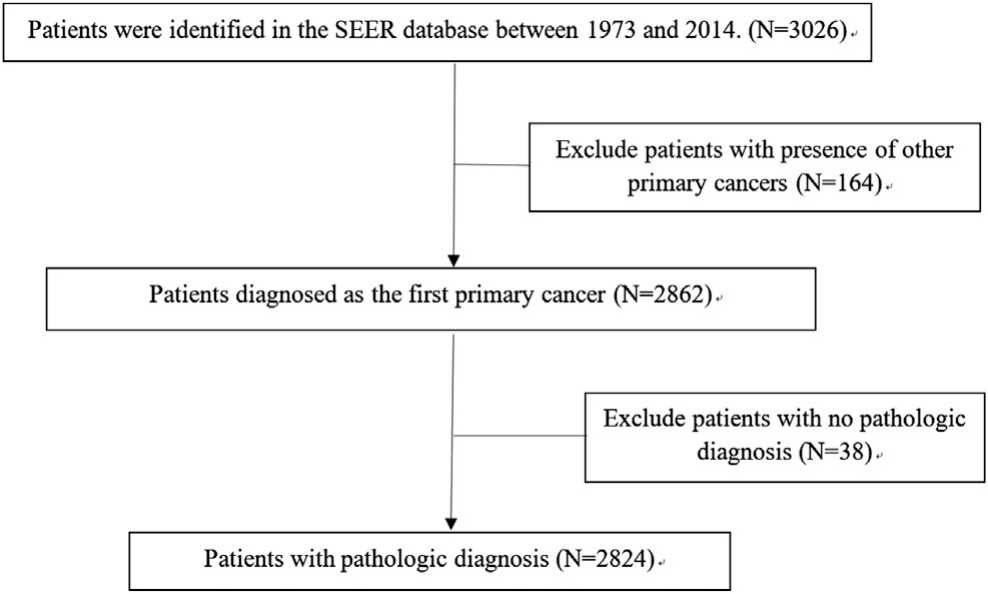
Screening for patients with medulloblastoma in the SEER database.

A total of 2,824 patients were identified from the SEER database as suitable for inclusion in the study and they were grouped as the training cohort (Figure 1). An additional 161 patients with complete data from SYSUCC database were identified and used as the validation cohort. The clinicopathological characteristics of patients in both cohorts are listed in Table 1.

**TABLE 1:** Clinicopathological variables of training and validation cohort.

Variables	Training cohort (*n* = 2,824)		Validation cohort (*n* = 161)		
	No	%	No	%	*p*-Value
Histopathology					
DMB	299	10.6	21	13.1	0.280
MB, NOS	2,439	86.4	138	85.7	
LC	86	3.0	2	1.2	
Surgery					
Total resection	1,293	45.8	80	49.7	<0.001
Subtotal resection	570	20.2	18	11.2	
Surgery, NOS	828	29.3	63	39.1	
No evidence	133	4.7	0	0.0	
Radiotherapy					
Yes	2,197	77.8	113	70.2	0.025
No evidence	627	22.2	48	29.8	
Chemotherapy					
Yes	1,864	66.0	110	68.3	0.546
No evidence	960	34.0	51	31.7	
Size (cm)					
≤6.5	1,718	60.8	57	35.4	<0.001
>6.5	43	1.5	1	0.6	
Unknown	1,063	37.7	103	64.0	
Dissemination					
Yes	168	5.9	8	5.0	0.586
No evidence	2,656	94.1	153	95.0	
Age					
Median (years)	14.2	—	12.0	—	0.001
≤3	580	20.5	15	9.3	
>3	2,244	79.5	146	90.7	
Sex					
Male	1,753	62.1	105	65.2	0.424
Female	1,071	37.9	56	34.8	

### Univariate and Multivariate Analyses

**FIGURE 2:**
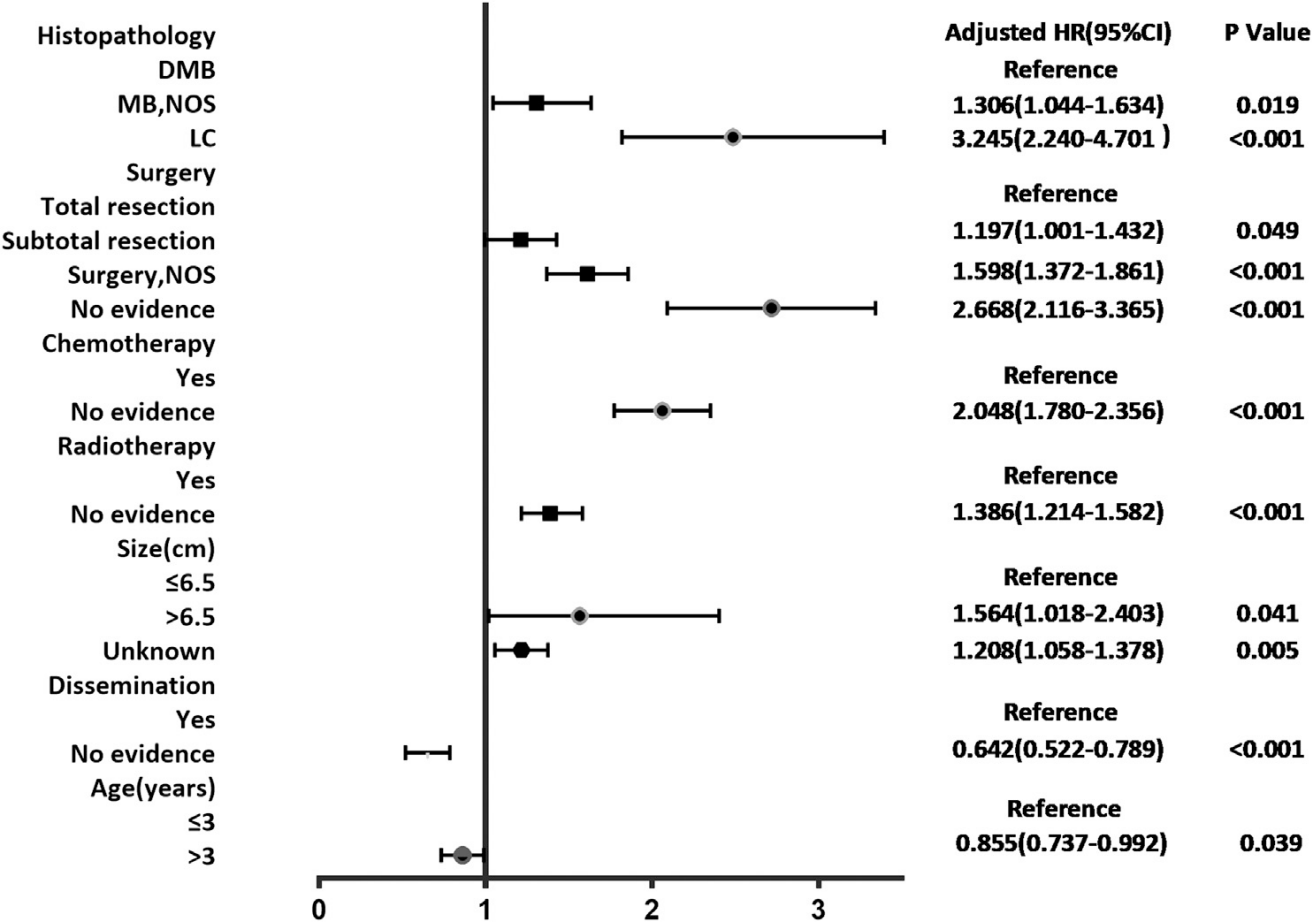
Forest plot of Cox proportional hazard ratio (HR) and 95% CI for overall survival of patients with medulloblastoma.

According to the cox regression analyses, we identified histopathology, surgery, radiotherapy, chemotherapy, tumor size, dissemination, and age of patients as independent factors correlated with the 1-, 3-, and 5-year OS rates in the training cohort (Table 2). No significant difference between prognosis and sex was observed. Forest plot was used to illustrate the effect and contribution of each independent prognostic factor in terms of hazard ratio (Figure 2).

**TABLE 2:** Univariate and multivariate Cox regression analysis of variables in the training cohort.

Variables	Univariate analysis		Multivariate analysis	
	HR (95% CI)	*p*-Value	HR (95% CI)	*p*-Value
Histopathology				
DMB	Reference	—	Reference	—
MB, NOS	1.344 (1.078–1.676)	0.009	1.306 (1.044–1.635)	0.019
LC	1.688 (1.247–2.284)	0.001	3.245 (2.240–4.701)	<0.001
Surgery				
Total resection	Reference	—	Reference	0.046
Subtotal resection	1.240 (1.037–1.483)	0.018	1.197 (1.001–1.432)	0.049
Surgery, NOS	1.859 (1.619–2.133)	<0.001	1.598 (1.372–1.861)	<0.001
No evidence	4.100 (3.245–5.179)	<0.001	2.668 (2.116–3.365)	<0.001
Chemotherapy				
Yes	Reference	—	Reference	—
No evidence	1.705 (1.514–1.919)	<0.001	2.048 (1.780–2.356)	<0.001
Radiotherapy				
Yes	Reference	—	—	—
No evidence	2.225 (1.954–2.533)	<0.001	1.386 (1.214–1.582)	<0.001
Size (cm)				
≤6.5	Reference	—	Reference	—
>6.5	1.916 (1.239–2.964)	0.003	1.564 (1.018–2.403)	0.041
Unknown	1.585 (1.405–1.787)	<0.001	1.208 (1.058–1.378)	0.005
Dissemination				
Yes	Reference	—	Reference	—
No evidence	0.591 (0.483–0.725)	<0.001	0.642 (0.522–0.789)	<0.001
Age (years)				
≤3	Reference	—	Reference	—
>3	0.684 (0.597–0.784)	<0.001	0.855 (0.737–0.992)	0.039
Sex				
Male	Reference	—	—	—
Female	0.982 (0.870–1.107)	0.762	—	—

### Nomogram

**FIGURE 3:**
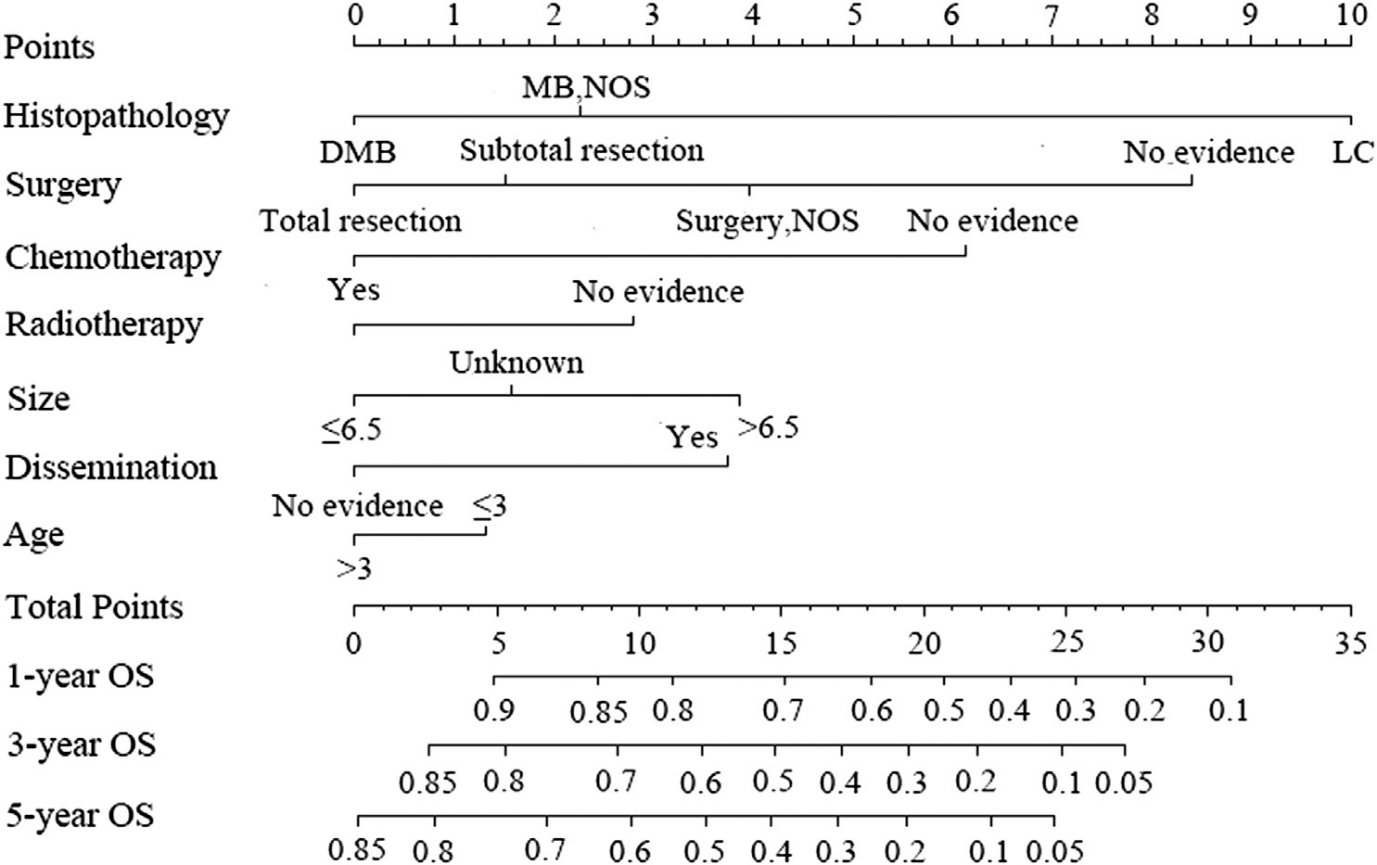
Nomogram predicting the 1-, 3-, and 5-year overall survival rates for patients with medulloblastoma.

**FIGURE 4:**
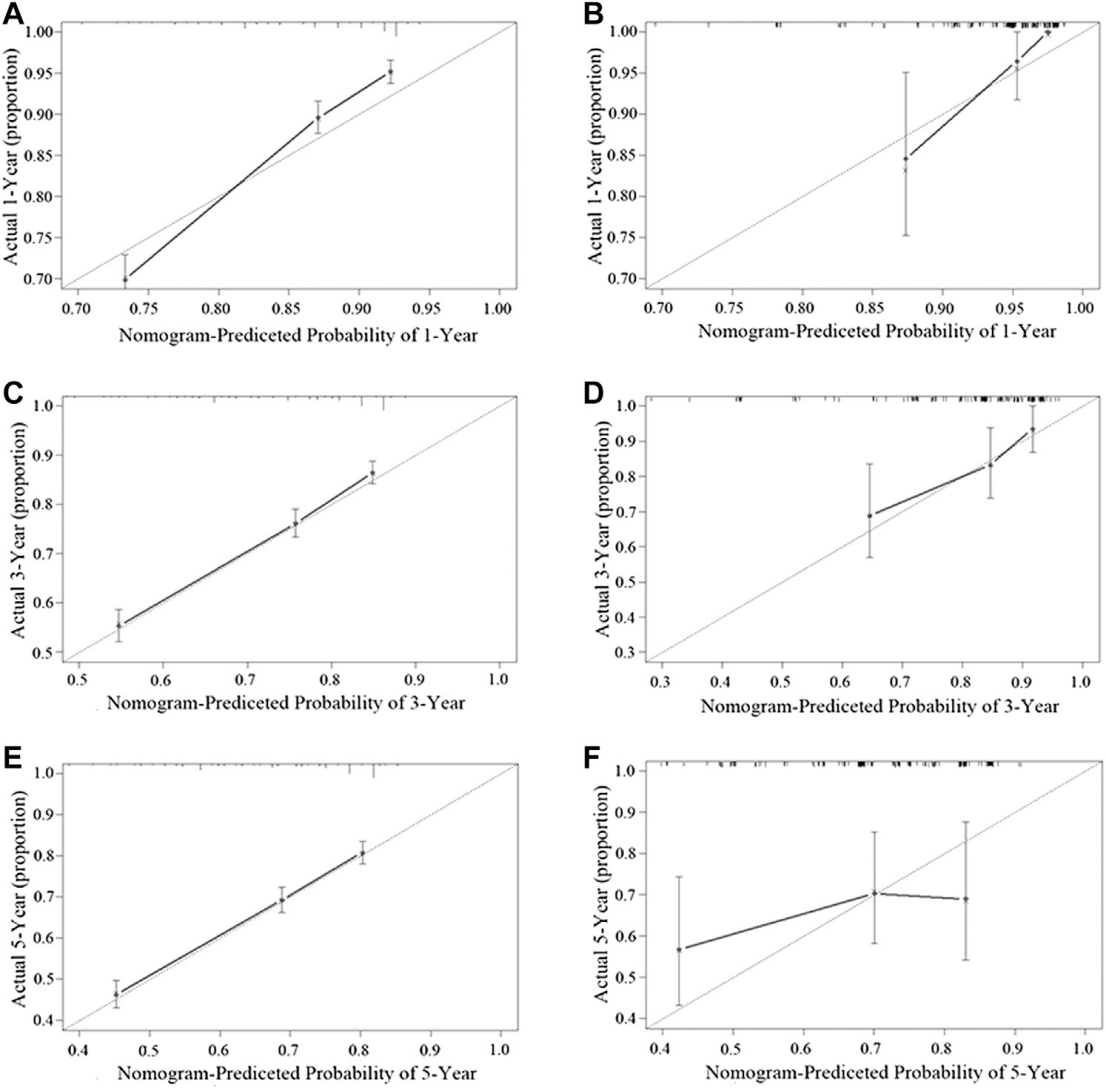
Performance of the nomogram in the training cohort **(A,C,E)** and the validation cohort **(B,D,F)** was evaluated by the calibration of the nomogram by training cohort and validation cohort. **(A)**, **(C)**, and **(E)**, respectively, stand for the 1-, 3-, and 5-year overall survival rate of the SEER cohort, and **(B)**, **(D)**, and **(F)**, respectively, stand for the 1-, 3-, and 5-year overall survival rate of the SYSUCC cohort (Training cohort: 0.681, 95% CI: 0.663–0.699 Validation cohort: 0.644, 95% CI: 0.551–0.737).

Figure 3 shows the nomogram model established for the 1-, 3-, and 5-year OS rates based on the selected factors. Histopathology was the biggest influencing factor on prognosis, whereas age of patients had the least effect. Validation of the nomogram was performed using bootstrap analyses with 1,000 resamples; internal and external validation cohorts showed favorable discrimination of the nomogram with C-index of 0.681 (95% CI: 0.663–0.699) and 0.644 (95% CI: 0.551–0.737), respectively, demonstrating that the nomogram could be clinically implemented. Internal and external calibration plots indicated high agreement between prediction and actual observation for the 1-, 3-, and 5-year OS rates (Figure 4).

### Nomogroups of Risk Grouping

**FIGURE 5:**
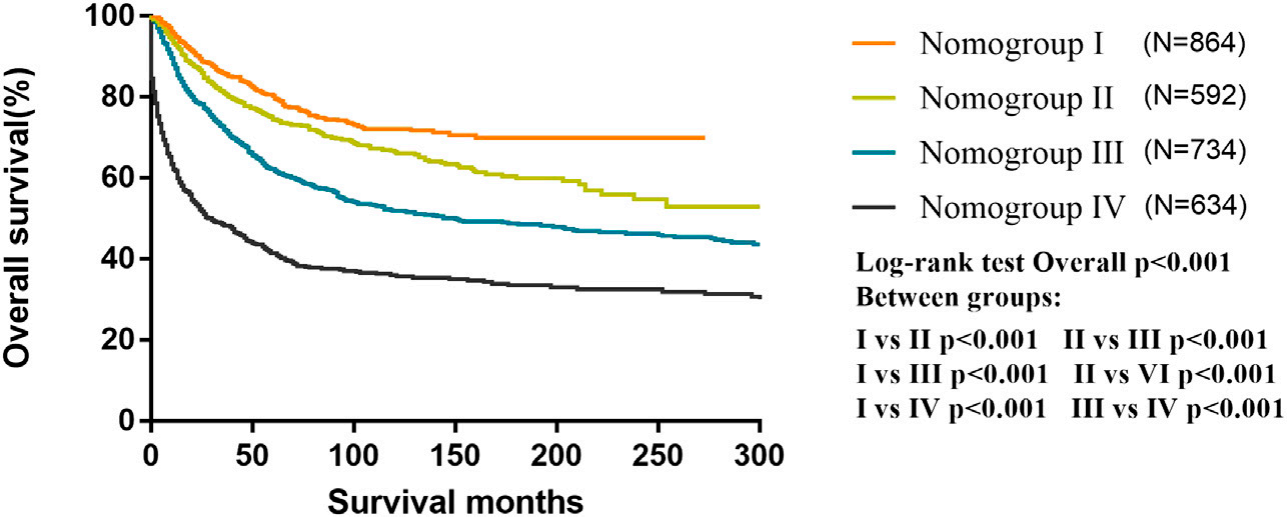
Kaplan–Meier curves for overall survival of the different risk nomogroups in the training cohort.

All patients were classified into four risk nomogroups according to the total score of each factor obtained from the nomogram of the training cohort. The nomogroups of the risk grouping were as follows: nomogroup I, score of 0–3.8 points; nomogroup II, 3.9–6.8 points; nomogroup III, 6.9–11.2 points; and nomogroup IV, ≥11.3 points. As shown in Figure 5, the prognosis between four risk nomogroups and the 5-year OS rate found by Kaplan–Meier analysis was 83.9%, 76.5%, 64.5%, and 46.8%, respectively, with statistically significant differences.

**FIGURE 6:**
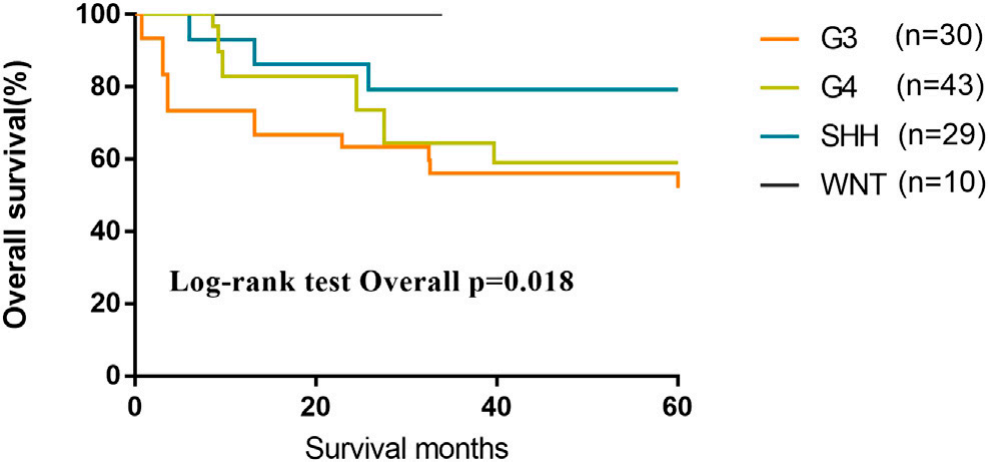
Kaplan–Meier curves of different subgroups for overall survival in the validation cohort.

We evaluated 116 patients from the database of SYSUCC to confirm the molecular subgrouping, and 112 MB patients were successfully subgrouped and diagnosed according to the revised WHO classification for brain tumors (2016). As of the last follow-up, the 5-year OS of patients of G3 and G4 subgroups were lower than those of Wnt and SHH subgroups (OS, Wnt 100%; SHH 79.3%; G4 59.1%; G3 56.1%; *p* = 0.018) (Figure 6).

## Discussion

As the most common tumor in pediatric central nervous system, MB accounts for 20–25% of all pediatric primary central nervous system tumors, which is classified as WHO grade IV tumor (Srikantha et al., 2010). Although recent studies have indicated that around 60% of MB patients could be curable, 10–15% of patients die within 2 years after the diagnosis (National Cancer Institute, 2002), indicating that the prognosis varied greatly in different cases. Based on the histopathological features, MBs are classified as classic medulloblastoma, large cell/anaplastic medulloblastoma, desmoplastic/nodular medulloblastoma, and medulloblastoma with extensive nodularity (Fuller and Scheithauer, 2007; Louis et al., 2007; Gilbertson and Ellison, 2008; Quinlan and Rizzolo, 2017). With intensive treatment, such as combined high-dose IV methotrexate and conventional chemotherapy combined with high-dose methotrexate or repeated cycles of myeloablative chemotherapy, the 5-year PFS ranged from 78.6 to 90% in patients with the desmoplastic/nodular and medulloblastoma with extensive nodularity subgroups. However, the severe toxicity of medication required more precise prognostic models to stratify different risk. A study demonstrated that the presence of metastatic disease at presentation, p53, TrkC, and ErbB2 expression in immunohistology were four predictors of overall survival of MB (Srikantha et al., 2010). Other studies suggested that molecular variants of diseases characterized by their gene expression with distinct clinicopathologic and molecular features, named WNT, SHH, G3, and G4, were significantly better associated with prognosis. The prognosis of patients of WNT subgroup has the best prognosis with greater than 90% of 5-year event-free survival. The prognosis of SHH-activated and G4 medulloblastomas is worse than WNT, with 5-year OS rate of 75% and 70%, respectively, whereas that of G3 has the most unfavorable 5-year overall of 50%. Although these four molecular subgroups have become effective predictors and important to divide MB patients into different risk stratification than histology pathology, they are not used comprehensively for all the institutions. In this study, the data of molecular detection were not shown in the SEER database. However, we detected the molecular subgroup in our MB patients as the validation cohort. The survival of SHH, WNT, and G3 in our research was similar to previous research, but the prognosis of G4 in our research was lower than other previous studies. The reason for the poorer G4 survival may be that there were 14.0% cases in G4, which did not receive the standard radiation and systemic chemotherapy as adjuvant therapy in comparison to other subgroups.

Chang staging for MB was introduced in 1960s, which classified MB patients into M0, M1, M2, M3, or M4 and T1, T2, T3a, T3b, or T4 according to their clinical features (Dufour et al., 2012). Although T stage was proved to be not associated with prognosis, M staging is still used for current risk stratification with 5-year OS rate of 47, 51, and 42% from M1 to M3 patients (Quinlan and Rizzolo, 2017). A set of clinical characteristics including the above M staging have classified patients into average-risk and poor-risk groups based on age of onset, extent of tumor resection, and extent of spreading at presentation (Gajjar et al., 2006; Rutkowski et al., 2010). Poor-risk patients were defined as patients with at least one of the following features: age less than 3 years, residual tumor ≥1.5 cm after maximal safe resection, and histopathological features of large cell or anaplastic MB. The average-risk patients were described as the patients without any of the above features (Quinlan and Rizzolo, 2017). The OS rates for poor-risk patients are 30–40%; for average-risk patients, the OS rates increase to 70–80% (Packer et al., 1999).

In clinical practice, the most commonly used prognosis system is the four molecular subgroup and the high-risk/average-risk system. However, the inconvenience of molecular detection approaches in daily clinical practice limited the application and prognostic evaluation in the majority of centers. However, the poor-risk/average-risk system is inefficient for stratifying patients into accurate prognosis. Biologic and clinical heterogeneity should be considered to stratify different treatment regimen. However, several clinical trials including SJYC07 presented negative risk-adapted therapy, which only confirmed that “reduced-intensity” therapy benefits a subtype SHH in early childhood medulloblastoma that would improve the progression-free survival. Because the aggressiveness, biological characteristics of individual tumors, and the efficacy of the treatment varied from patient to patient, establishing a better stratified and individualized prognostic model would provide a much more significant experience to both clinicians and patients.

However, to the best of our knowledge, there was no nomogram available for the evaluation of MB. The objective of our study is to establish a novel, convenient comprehensive nomogram model to evaluate OS for MB patients. We develop the individual prognostic nomogram from the SEER data set and validate an independent data set from SYSUCC database. Our study shows the value of clinical characteristics predictor of individualized MB prognosis with the nomogram model. The nomograms showed precise and stable prediction when analyzing both the training and the validation cohorts, indicating its good clinical applicability. The traditional prognostic model included the clinical parameters to divide patients into two levels as the poor-risk and average-risk system, which is insufficient for precise prediction. Compared to the previous model, the MB cohort was satisfactorily separated into four distinct prognostic groups with the cutoff point of 3.9, 6.9, and 11.3 in our nomogram model.

According to our nomogram, if an MB patient would have histopathology of LC, received radiotherapy, chemotherapy, and total resection, tumor size greater than 6.5 cm, age older than 3 years, and without dissemination, his total score would be 13.9. Therefore, as shown in Figure 5, we could speculate that this patient was categorized as nomogroup IV with a 44% probability of 5-year OS. In this case, clinician should consider close follow-up because of the poor prognosis.

Seven clinical and pathology factors in our model, including histopathology, surgery, radiotherapy, chemotherapy, tumor size, dissemination, and age, were independently correlated with the OS rates. First, different pathology subtypes may represent distinct biological behaviors of MB, which may affect further treatment strategies. Large-cell and anaplastic MB are regarded as high-risk disease, which demonstrates that patients have a poorer survival and should be received more intensive chemotherapy and higher dose of radiation. However, desmoplastic nodular MB may benefit from a better outcome, because desmoplastic nodular MB shared the best survival in the histopathology in our study, which was consistent with previous study (Packer et al., 1999). We still emphasize that the four main histologic types are essential to the prognostic prediction, although molecular subgroups seem to be more popular than histology subgroups. Second, standard treatment is the therapeutic stratification for medulloblastoma patients. Because relapses of MB are common in patients with nonstandard treatment, the maximal safe resection along with radiation and chemotherapy will benefit the long-term survival of MB patients. Third, recent publications have shown that T stage in the Chang’s operative staging system was not related to survival; however, our model indicated that tumor size could still predict outcome. Although size is not a common risk factor in previous prognostic classification, it is often difficult to separate the residual tumor from normal tissue surgically if the tumor has severe adhesion with large size, which also indicates more probability to have metastasis. As a result, patients with the presence of metastases (M1–M4) or residual disease >1.5 cm^2^ belong to the high-risk group according to North American stratification. Last but not least, similar high-risk stratification can be applied to younger MB patients younger than 3 years at diagnosis, who were not recommend to receive radiation instead of prolonged and intensive chemotherapy regimen with the aim of reducing the risk of relapse. As a result, prospective study or evidence from multiple databases is needed to confirm the staging system of MB.

There are several limitations in our study. First, this study was a retrospective analysis although we used reliable databases from the SEER system and that of SYSUCC to investigate the applicability of this nomogram in the Caucasian and Asian populations. Second, some details in the SEER database, such as the molecular subgroups, radiotherapy doses, and chemotherapy regimens, were not provided, and other promising prognostic parameters could not be analyzed. Third, information about several important clinicopathological parameters from SEER such as tumor size, tumor location, and histopathology were not complete. To guard against bias, we have to retain these data, which is defined “unknown or NOS.” Fourth, patient cases from the training and validation cohort spanned for nearly half a century, which may have caused certain statistical biases for not accounting advances in techniques of treatment and diagnosis.

## Conclusion

A nomogram, consisting of seven clinicopathological and treatment-related factors, for predicting the 1-, 3-, and 5-year OS of MB patients was constructed from a Caucasian cohort and was successfully validated in an Eastern database. This proposed survival prognostic model could be useful for differentiating patients of different risk groups to better provide individualized treatment decision and surveillance.

## Data Availability Statement

The datasets presented in this study can be found in online repositories. The names of the repository/repositories and accession number(s) can be found in the article/supplementary material.

## Ethics Statement

Our study was approved by the Ethics Committee of Sun-Yat-Sen University Cancer Center Health Authority (identifier: B2020-199-01). Written informed consent from the participants’ legal guardian/next of kin was not required to participate in this study in accordance with the national legislation and the institutional requirements.

## Author Contributions

All authors participated in this research, including conception and design (CG, DY, XL, YM, and QY), data acquisition (CG, XL, and HH), data analysis and interpretation (CG, DY, and XL), material support (JZ, FL, and XL), study supervision (CG, DY, YM, and QY), as well as drafting the article or critically revising (CG, DY, XL, HH, JZ, FL, YM, and QY). The final version is ensured and approved by all authors.

## Funding

This research was supported by the Science and Technology Planning Project of Guangdong Province (2017A020215030 and 2014A020212098).

## Conflict of Interest

The authors declare that the research was conducted in the absence of any commercial or financial relationships that could be construed as a potential conflict of interest.
